# Congenital Pulmonary Malformation in Children

**DOI:** 10.6064/2012/209896

**Published:** 2012-05-08

**Authors:** Montasser Nadeem, Basil Elnazir, Peter Greally

**Affiliations:** Paediatric Respiratory Department, The Adelaide and Meath Hospital, Dublin, Incorporating the National Children's Hospital (AMNCH), Tallaght, Dublin 24, Ireland

## Abstract

Congenital Pulmonary Malformations (CPMs) are a group of rare lung abnormalities affecting the airways, parenchyma, and vasculature. They represent a spectrum of abnormal development rather than discrete pathological entities. They are caused by aberrant embryological lung development which occurs at different stages of intrauterine life.

## 1. Congenital Pulmonary Malformations (CPMs)

CPMs are a group of rare lung abnormalities affecting the airways, parenchyma, and vasculature. They represent a spectrum of abnormal development rather than discrete pathological entities. They are caused by aberrant embryological lung development which occurs at different stages of intrauterine life.

With improved resolution of foetal sonography and Doppler studies, many of these lesions are detected “in utero.” Their natural history can be quite variable. Lesions may resolve prior to birth. Some CPMs may present in the early neonatal period with respiratory distress due to a mass effect, while others may be asymptomatic only to be detected in later life on CXRs performed for other reasons. Certain forms of CPM have the potential to undergo malignant change. Conversely, pleuropulmonary blastoma may masquerade as some forms of CPM. When lesions are symptomatic in early life there can be no doubt that surgical resection is the only option. However, the decision-making process becomes more complex when clinicians encounter asymptomatic lesions, in healthy infants, which have been detected either by antenatal USS or incidental chest radiograph.

The presentation, natural history, diagnosis and management of congenital pulmonary airway malformation, congenital lobar emphysema, pulmonary sequestration, bronchogenic cyst, and pleuropulmonary blastoma will be discussed.

## 2. Congenital Pulmonary Airway Malformation (CPAM)

Congenital pulmonary airway malformation (CPAM) was previously known as congenital cystic adenomatoid malformation (CCAM). Histologically, the lesion of CPAM is characterised by solid adenomatous areas, which consist of closely packed tubular structures resembling terminal bronchioles without mature alveoli [1]. These areas closely resemble normal foetal lung at 16-week gestation [2]. Interspersed with these adenomatous areas are cysts. Postnatally the downstream alveoli can only be ventilated collaterally via the pores of Kohn.

The incidence of CPAM is 1 per 8300 to 35,000 [3], Table 1. It usually affects a single lobe and it is no association with sex predilection [1]. There are five subtypes of CPAM (0–4) depending on the proportion of cysts and adenomatous tissue and the dominant cell types [4]. Types 1 and 2 occur with most frequency [4, 5]. Type 1 lesions are usually unicystic or paucicystic and may contain fluid, they contain little or no adenomatous component. The cysts are greater than 2 cm in diameter and lined with pseudostratified columnar epithelium [4]. Type 2 lesions contain more uniform small cyst of less than 1 cm in diameter [4]. Mucous cells and cartilage are seen in type 1, but not type 2 [4]. Smooth and striated muscle fibres are seen in types 1 and 2, respectively [4]. A coexistent CPAM type 2 has been reported in half of the patients with extralobar sequestration (ELS) [6], however the separation from the lung and the presence of a systemic blood supply are helpful to distinguish between these entities [3]. Type 4 lesions contain large cysts lined with flat alveolar cells, some of which contain surfactant [7]. They involve more peripheral lung. They can present with tension pneumothorax [7]. Type 4 CPAM was considered within type 1, in older publications, as both types are composed of large cysts (up to 10 cm in diameter) [8]. Large cysts are usually observed and pneumothorax can occur in Type 1 PPB [3], which has a histological overlap with type 4 CPAM. It is not easily possible to differentiate between both entities on histology alone [9–11].

### 2.1. Natural History

 The natural history of CPAM can be quite variable. Spontaneous regression during the course of gestation is not uncommon. In 29 patients with CPAM, during fetal life, the size of the lesion decreased in 8 patients, of whom 7 required postnatal surgical resection, and disappeared in 4 patients, of whom 3 required postnatal surgical resection [17]. In children with disappearing fetal lung masses, postnatal CT scans are needed to exclude the persistent abnormalities, which are often subtle or not apparent on plain radiographs [18].

### 2.2. Prenatal versus Postoperative Pathological Diagnoses

With improved resolution of foetal sonography, many of the CPAM lesions are detected “in utero.” However appropriate postnatal investigations, rather than antenatal diagnosis, are essential for surgical decision [19]. No correlation between antenatal ultrasound features and histological diagnosis after surgery has been reported [19, 20]. In one hundred and five complete records of asymptomatic children with prenatally diagnosed lung lesions, the postoperative pathologic diagnoses were different from preoperative radiological findings in 9 patients [21]. In a study of 17 fetuses diagnosed with CPAM by prenatal ultrasound, pathological diagnosis was confirmed in 57% of those with known pathological diagnosis [20]. Tsai et al. showed that, in one hundred and five complete records of asymptomatic children with prenatally diagnosed lung lesions, the postoperative pathologic diagnoses were different from preoperative radiological findings in 9 patients [21].

### 2.3. Clinical Presentation

 CPAM may present in the early neonatal period with respiratory distress due to a mass effect, while others may be asymptomatic only to be detected in later life on CXRs performed for other reasons. Sauvat et al., reported that 3 out of 29 patients with CPAM experience symptoms during the first weeks of life [17]. As many as 86% (18 out of 21) of patients, who were asymptomatic at birth, had become symptomatic by 13 years of age (median age of 2 years) [22]. In patients with CPAM, pneumonia with or without infected CPAM was reported in 43%, respiratory distress in 14%, and spontaneous pneumothorax in 14% [22]. In 31 patients with bronchopulmonary malformations CPAM (*n* = 13), pulmonary sequestrations (*n* = 6), bronchogenic cysts (*n* = 3), congenital lobar emphysemas (*n* = 9), Shanmugam et al. reported that respiratory distress, respiratory infections/pneumonias, and dyspnoea occurred in 9, 22, and 9 out of these 31 patients, respectively [23]. In 16 patients with CPAM, neonatal respiratory impairment, pneumothorax, and recurrent respiratory tract infections occurred in 12, 1, and 3 patients, respectively [24].

### 2.4. Postnatal Diagnosis

 The diagnosis is suggested by CXR, however, a thoracic CT scan is required to confirm the diagnosis [25, 26]. Winters et al. reported that CXR features can be subtle in children with disappearing fetal lung masses, hence postnatal CT scan is needed [18]. In 29 patients with CPAM, postnatal X-ray and CT scan were abnormal in 17 and 25, respectively [17]. CPAM lesions may be confused for a congenital diaphragmatic hernia. Heij et al. showed that 4 out of 16 patients with CCAM had laparotomy for presumed diaphragmatic hernia [24]. In these circumstances, CT scan can be helpful.

### 2.5. Is There Association between CPAM and Malignancy?

 An association between type 1 CPAM and bronchoalveolar carcinoma has been reported [27]. In patients with type 1 CPAM, Langston showed that microscopic foci of bronchoalveolar carcinoma and focal mucous cell hyperplasia could occur in up to 5/16 and 2/16 of patients [10]. In those with type 4 CPAM, focal stromal hypercellularity and pleuropulmonary blastoma were documented in 4/8 and 1/8 of patients [10]. The distinction between type 4 CPAMs and grade 1 pleuropulmonary blastomas may not be possible on histology alone [9–11]. Primary pulmonary rhabdomyosarcoma (RMS) has been reported in 13-month- old boy with CPAM [28].

### 2.6. Maternal Steroid and Fetal Therapy

In 13 patients with predominantly microcystic CPAM, Curran et al. showed that fetuses, who had CPAM volume-to-head ratio >1.6 or nonimmune hydrops, experience an improvement following prenatal betamethasone [29]. In these patients, hydrops and CPAM volume-to-head ratio improved in 77.8% and 61.5% of the patients, respectively. Adzick reported two fetuses (>32-week gestation), with lung lesion and hydrops, experienced an ex utero intrapartum therapy strategy with resection of the lesion and survived [30]. In fetuses with CPAM and hydrops, fetal resection or thoracoamniotic shunt is recommended [31]. Serial fetal thoracocenteses may be an alternative or an adjunct to fetal surgery in selected cases [31]. Adzick et al. showed that hydrops resolution and neonatal survival occurred in 8 out of 13 fetuses, with CPAM and nonimmune hydrops, who experienced fetal surgical resection of the lesions at 21 to 29 week of gestation [32]. Five out of six fetuses, with a very large solitary cyst, who had thoracoamniotic shunting, survived [32]. In 67 fetuses with cystic lung lesions, percutaneous intrauterine laser therapy and thoracoamniotic shunts had been performed in 1 and 3 fetuses, respectively [20].

### 2.7. Recommendation and Management

Air travel in children suffering from cystic lung lesions is controversial because of the risk of pneumothorax. Most clinicians caution against air travel in children with enlarging cystic lesions. All infants with antenatally diagnosed CPAM should be evaluated. Symptomatic children require surgical resection [33]. When CPAM is asymptomatic some clinicians prefer to observe rather than refer for surgery. However, when one considers the risk of pulmonary compression, infection, and the low risk for malignancy, it is not surprising that many clinicians prefer the operative approach [23, 33]. In the majority of cases, pulmonary resection is indicated as soon as the diagnosis is made, with emergency resection in those with severe respiratory distress.

## 3. Bronchogenic Cyst (BC)

Bronchogenic cyst is a rare CPM, with a prevalence rate of 1 per 68,000 [14]. BC represents part of the spectrum of broncho-pulmonary foregut malformations [34]. The primitive foregut gives rise through a central outpunching to the trachea-bronchial tree. Between the 5–16th week of gestation the bronchi develop by a process of budding and branching. Buds can develop at any site along the trachea-bronchial tree which, if their development arrests, become BC. The cysts contain tissue normally found in the airways (mucous glands, smooth muscle, elastic tissue, and cartilage). BC may occur in paratracheal, carinal, paraesophageal, hilar, suprasternal notch, and miscellaneous locations [35–37]. The latter occurs when buds detach and migrate to ectopic sites, for example, pericardial, cervical, and abdominal [35–37].

### 3.1. Diagnosis and Presentation

 BC may be detected as incidental findings on chest radiographs and account for 10% of mediastinal masses in children. The postnatal diagnosis of BC may be suspected on chest radiograph but a thoracic CT scan is needed to confirm the diagnosis [38]. In a case series of 33 patients with bronchogenic cysts, the lesions usually presented as spheroid mediastinal masses, near the carina or right paratracheal area [39]. Newborns with large cysts can develop respiratory distress, cyanosis, and feeding difficulty [40]. Compression of the trachea and respiratory arrest has been reported in infant with BC [41]. BC in a bronchial location may present with wheeze and recurrent pneumonia. If the airway obstruction is only partial and gives rise to a ball-valve effect, then hyperinflation of the distal lung will occur which may mimic CLE. Where the obstruction is complete, atelectasis of the distal lung may occur leading to infection. High tracheal BC may present with stridor. Cysts may communicate with the airway and become infected and rupture. Paraesophageal cyst may give rise to dysphagia. Pericardial BC may present with Superior Vena Cava obstruction. In a series of 10 infants and children (age between 16 days and 6 years) with bronchogenic cyst, respiratory distress, cyanosis, chronic cough, and fever and dysphasia were reported in 70%, 40%, 50%, and 20% of patients, respectively [42]. The differential diagnosis depends on location and may include lymphadenopathy, oesophageal duplication cyst, neuroblastoma, cystic hygroma, and dermoid cyst.

### 3.2. Association between BC and Malignancy

A rare association between BC and neuroblastoma had been reported in 2 children [43]. A recent meta-analysis of bronchogenic cysts found that 45% of 683 asymptomatic adults progressed to develop complications and that there was a small risk 0.7% of malignant transformation within the cyst [44]. Bronchoalveolar cell carcinoma is the most common form of malignancy associated with BC.

### 3.3. Management

Surgical excision should be considered in all cases because of the likelihood of eventual development of symptoms and malignant change [45].

## 4. Congenital Lobar Emphysema (CLE)

CLE is relatively rare affecting 1 in 20,000 live births [12]. It occurs more commonly in males [13] and most frequently affects the left upper lobe [46]. Most series report that within the affected lobe the alveoli are anatomically and numerically normal. It is postulated that the condition is caused by either absence or maldevelopment of cartilaginous rings or bronchomalacia of a proximal airway due to extrinsic compression “in utero.” However, in 50% of cases the airway appears normal. The net effect is air trapping in the affected lobe [47].

### 4.1. Presentation and Diagnosis

 Lesions may be asymptomatic or present with respiratory distress in the newborn period [48]. Later, infants may experience dyspnoea (57%) or recurring respiratory infection (28%) [48]. On CXR, hyperlucency of the affected lobes is the characteristic feature [48]. The diagnosis may be confirmed on chest CT [49]. The differential diagnosis includes type 1 unicystic CPAM, pneumtothorax, Swyer-James syndrome, bronchogenic cyst, and diaphragmatic hernia.

### 4.2. Association with Malignancy and Heart Diseases 

Type I pleurapulmonary blastoma may mimic CLE. In one report of a patient with asymptomatic CLE, the lesion was resected due to increasing size and pleuro-pulmonary blastoma type I was confirmed on histology [50]. This overlap in the spectrum of diagnoses prompts the surgical view to resect CPM. A cardiac evaluation is required because as many as 15% of cases may have congenital heart disease [48].

### 4.3. Management

 Early surgical excision is required for newborns with respiratory distress [49, 51]. However, infants and older children who are asymptomatic or have minimal symptoms can be treated conservatively [52]. Surgical excision of the affected lobe is the appropriate treatment in all infants under 2 months of age and in infants older than 2 months presenting with severe respiratory symptoms. Infants older than 2 months presenting with mild to moderate respiratory symptoms associated with normal bronchoscopic findings can be treated conservatively [48].

## 5. Bronchopulmonary Sequestration (BPS)

BPS is a rare congenital malformation of the lower respiratory tract and comprises 0.15 to 6.4% of all CPM. The incident rate of pulmonary sequestration is 0.29% [15]. It is an area of nonfunctioning lung tissue that commonly receives its arterial blood supply from the descending aorta. The sequestered lobe has no communication with the tracheobronchial tree. BPS is classified as either intralobar sequestration (ILS), in which the lesion is located within a normal lobe and lacks its own visceral pleura or extralobar (ELS), in which the lesion is located outside the lung and has its own visceral pleura.

ILS lesions account for 80% of sequestrations [16]. The sequestration commonly occurs in the lower lobe, primarily in the left posterior basal segment [16]. With respect to ILS, the venous drainage is usually to the pulmonary circulation [53]. However, ELS frequently have systemic venous drainage.

Other congenital anomalies occur in 3/18 and 3/4 of ILS and ELS, respectively [54]. In 28 patients with BPS, diaphragmatic hernia, atrial septal defect, dextrocardia, double superior caval vein, and a sliding hernia and an esophageal bronchus communicating with an ELS have been seen in 2 and 1 each, respectively [54]. Gezer et al. reported a case of diaphragmatic hernia in 1 (12.5%) patient with ELS [53]. In one series, the association between ELS and type 2 CPAM has been reported in as many as 50% of patient [6].

### 5.1. Prenatal and Postnatal Diagnosis

In a previous study of pregnant women with antenatally diagnosed ELS, the lesions regressed before delivery in 28/41 (68%) cases [32]. Postnatal diagnosis of ELS often can be made from chest radiograph appearance. However, ultrasonography with Doppler imaging or thoracic CT with contrast may be used to define the lesion. ELS are often discovered in infants who present with other conditions for example, heart failure, polyhydramnios, or prematurity.

## 6. Presentation

The age of presentation is variable in children with ELS. In a case series of 50 patients with ELS, 24% were diagnosed prenatally and 61% were diagnosed by three months of age [6]. In 27 patients (age between 3.5 and 51 years) with BPS, 10, 6, 2, and 2 experience recurrent pneumonia, chest pain, hemoptysis, and shortness of breath, respectively [53]. The adjacent lung is frequently bronchiectatic in resected ILS specimens. The differential diagnosis of BPS includes CPAM, congenital diaphragmatic hernia, bronchogenic cyst, and mediastinal tumour.

### 6.1. Complications

These include haemoptysis, haemothorax, and malignant transformation. Levine et al., documented severe congestive heart failure in 2 infants with BPS and large arterio-venous shunts [55]. A localised carcinoma in the sequestrated lung tissue has also been reported [56].

### 6.2. Fetal Therapy

In fetuses with BPS and hydrops, open fetal surgery, coagulation of the blood supply, or thoracoamniotic shunting has been recommended [57]. BPS with hydrops has been treated successfully with laser coagulation of the feeding systemic artery of the sequestration under ultrasound guidance [57]. Rammos et al. reported 2 babies, with BPS, who required postnatal thoracotomy performed on the 10th day of life in spite of prenatal amnioperitoneal shunt, pleural drainage, and laser ablation of the feeding artery [58].

### 6.3. Management

 Immediate surgical intervention is required in patients with respiratory distress. In view of the high rate of complications, elective resection is often recommended even in asymptomatic patients with BPS [54]. Ayed and Owayed reported that the age of intervention was 11 weeks (range, 13–43 weeks), in those with pulmonary sequestration [51].

## 7. Pleuropulmonary Blastoma (PPB)

It is a rare tumor of pleura and lung in young children. The incidence of PPB is 1 in 250,000 live births [59]. There are 3 different types of PPB. Type 1 lesion presents in a cystic form, which is indistinguishable from benign lung cyst. However, nodules within the cystic lesions and solid masses are present in types 2 and 3, respectively. PPB is the most frequent malignancy associated with childhood lung cysts. A quarter of PPBs are associated with a familial predisposition to dysplasia. PPB should be considered in infants and children presenting with lung cysts and pneumothorax, bilateral lung cysts, or a family history of PPB-associated conditions, including renal cystic disease and small bowel polyps. Cerebral metastases had been reported in types 2 and 3 PPB [60]. This suggests routine brain imaging to monitor development of metastasis in patients with PPB.

## 8. Conclusion

Congenital Lung malformations are becoming more frequently recognised with the advent of improved antenatal imaging. There is considerable debate among clinicians as to the best approach to these lesions in asymptomatic infants as many of the lesions have the potential to involute over time. Further longitudinal studies are needed to address the appropriate approach towards management for each of the CPM discussed.

## Figures and Tables

**Table 1:** Common congenital pulmonary malformations.

	Incidence/prevalence	Features and presentation	Treatment	Comments
CPAM	Incidence is 1 per 8300 to 35,000 [3]. No sex predilection [1].	Usually limited to one lobe. Many cases are detected by prenatal U/S. CPAM may present in the early neonatal period with respiratory distress. 86% of patients, who were asymptomatic at birth, had become symptomatic by 13 years of age (median age 2 years). Symptoms include pneumonia with or without infected CPAM, respiratory distress, and spontaneous pneumothorax.	Surgical resection is the definitive treatment. Asymptomatic patients can be observed with serial imaging.	Lesions regress antenatally in 59%. Type 4 and type 1 tend to carry a malignancy risk.

CLE	Prevalence rate of 1 in 20,000 live births [12]. Males > females [13].	The left upper lobe is affected in half of the cases. Lesions may be asymptomatic or present with respiratory distress in the newborn period. Those with the lesion may experience dyspnoea or recurring respiratory infection. 15% of cases may have congenital heart disease.	Resection of the affected lobe in symptomatic newborn. Conservative treatment in Asymptomatic patients.	Congenital cardiac anomalies often accompany CLE.

BC	Prevalence rate of 1 per 68,000 [14].	It can occur throughout the tracheobronchial tree. Large cyst presents with respiratory distress and cyanosis in newborns. BC may be detected as incidental findings on chest radiographs. Newborns with large cysts can develop respiratory distress, cyanosis, and feeding difficulty. Compression of the trachea and respiratory arrest has been reported. Wheeze, stridor, atelectasis of the distal lung, dysphagia, and recurrent pneumonia can occur.	Surgery is recommended in all cases.	Typically presented during the second decade of life.

BPS	Incident is 0.29% [15]. Male > female [16].	Lower lobes are mostly affected. Newborn may present with respiratory distress. Patients with ELS may remain asymptomatic. In a case series of 50 patients with ELS, 24% were diagnosed prenatally and 61% were diagnosed by three months of age. Recurrent pneumonia, chest pain, hemoptysis, and shortness of breath, respectively, can occur. The adjacent lung is frequently bronchiectatic in resected ILS specimens.	Symptomatic Patients require surgical intervention. Elective resection is recommended in asymptomatic patients with ILS.	Infection, heart failure, carcinoma, and bleeding can occur in sequestrated lung.

CPAM: congenital pulmonary airway malformation, CLE: congenital lobar emphysema, BC: bronchogenic cysts, BPS: bronchopulmonary sequestration, ILS: intralobar sequestration, ELS: extralobar sequestration.

## Abbreviations

CPAM: Congenital pulmonary airway malformation
CLE: Congenital lobar emphysema
BC: Bronchogenic cysts
BPS: Bronchopulmonary sequestration
PPB: Pleuropulmonary blastoma.
